# Supplementary material for: Resolving the full spectrum of human genome variation using Linked-Reads
Source: Genome Res. 2019 Apr;29(4):635–45. doi: 10.1101/gr.234443.118 (PMC6442396; doi:10.1101/gr.234443.118)
Supplement: Supplemental Material [file supp_gr.234443.118_Supplemental_longranger-2.2.zip › longranger-2.2.tar.bz2!/lr2.2-public/lib/stan/cmdstan/stan/doc/api/html/segment_8hpp_source.html]

Stan: src/stan/math/matrix/segment.hpp Source File


|  |  |
| --- | --- |
|  | Stan  2.5.0  probability, sampling & optimization |


- Main Page
- Related Pages
- Namespaces
- Classes
- Files

- File List
- File Members

All Classes Namespaces Files Functions Variables Typedefs Enumerations Enumerator Friends Macros Pages

- src
- stan
- math
- matrix

segment.hpp

Go to the documentation of this file.

1 #ifndef STAN\_\_MATH\_\_MATRIX\_\_SEGMENT\_HPP

2 #define STAN\_\_MATH\_\_MATRIX\_\_SEGMENT\_HPP

3

4 #include <stan/math/matrix/Eigen.hpp>

5 #include <vector>

6 #include <stan/math/error\_handling/check\_greater.hpp>

7 #include <stan/math/error\_handling/check\_less\_or\_equal.hpp>

8

9

10 namespace stan {

11  namespace math {

12

17  template <typename T>

18  inline

19  Eigen::Matrix<T,Eigen::Dynamic,1>

20  segment(const Eigen::Matrix<T,Eigen::Dynamic,1>& v,

21  size\_t i, size\_t n) {

22  stan::math::check\_greater("segment(%1%)",i,0.0,"n",(double\*)0);

23  stan::math::check\_less\_or\_equal("segment(%1%)",i,static\_cast<size\_t>(v.rows()),"n",(double\*)0);

24  if (n != 0) {

25  stan::math::check\_greater("segment(%1%)",i+n-1,0.0,"n",(double\*)0);

26  stan::math::check\_less\_or\_equal("segment(%1%)",i+n-1,static\_cast<size\_t>(v.rows()),"n",(double\*)0);

27  }

28  return v.segment(i-1,n);

29  }

30

31  template <typename T>

32  inline

33  Eigen::Matrix<T,1,Eigen::Dynamic>

34  segment(const Eigen::Matrix<T,1,Eigen::Dynamic>& v,

35  size\_t i, size\_t n) {

36  stan::math::check\_greater("segment(%1%)",i,0.0,"n",(double\*)0);

37  stan::math::check\_less\_or\_equal("segment(%1%)",i,static\_cast<size\_t>(v.cols()),"n",(double\*)0);

38  if (n != 0) {

39  stan::math::check\_greater("segment(%1%)",i+n-1,0.0,"n",(double\*)0);

40  stan::math::check\_less\_or\_equal("segment(%1%)",i+n-1,static\_cast<size\_t>(v.cols()),"n",(double\*)0);

41  }

42

43  return v.segment(i-1,n);

44  }

45

46

47  template <typename T>

48  std::vector<T>

49  segment(const std::vector<T>& sv,

50  size\_t i, size\_t n) {

51  stan::math::check\_greater("segment(%1%)",i,0.0,"i",(double\*)0);

52  stan::math::check\_less\_or\_equal("segment(%1%)",i,sv.size(),"i",(double\*)0);

53  if (n != 0) {

54  stan::math::check\_greater("segment(%1%)",i+n-1,0.0,"i+n-1",(double\*)0);

55  stan::math::check\_less\_or\_equal("segment(%1%)",i+n-1,static\_cast<size\_t>(sv.size()),"i+n-1",

56  (double\*)0);

57  }

58  std::vector<T> s;

59  for (size\_t j = 0; j < n; ++j)

60  s.push\_back(sv[i + j - 1]);

61  return s;

62  }

63

64  }

65 }

66 #endif

stan::math::check\_greater

bool check\_greater(const char \*function, const T\_y &y, const T\_low &low, const char \*name, T\_result \*result)

**Definition:** check\_greater.hpp:58

check\_greater.hpp

check\_less\_or\_equal.hpp

Eigen.hpp

stan::math::check\_less\_or\_equal

bool check\_less\_or\_equal(const char \*function, const T\_y &y, const T\_high &high, const char \*name, T\_result \*result)

**Definition:** check\_less\_or\_equal.hpp:52

stan::math::segment

Eigen::Matrix< T, Eigen::Dynamic, 1 > segment(const Eigen::Matrix< T, Eigen::Dynamic, 1 > &v, size\_t i, size\_t n)

Return the specified number of elements as a vector starting from the specified element - 1 of the sp...

**Definition:** segment.hpp:20


---

[ Stan Home Page ]

*© 2011–2014,
Stan Development Team.*
